# Supplementary material for: A UMLS-based spell checker for natural language processing in vaccine safety
Source: BMC Med Inform Decis Mak. 2007 Feb 12;7:3. doi: 10.1186/1472-6947-7-3 (PMC1805499; doi:10.1186/1472-6947-7-3)
Supplement: Additional file 13 — RAP application directory. Sets up directory for RAP (RDF application for PHP) source code [file 1472-6947-7-3-S13.gz › rap/doc/phpdoc/__filesource/fsource_default__constants.php.html]

xml version="1.0" encoding="iso-8859-1"?


File Source for constants.php


# Source for file constants.php

Documentation is available at constants.php

```
1. <?php
2. // ----------------------------------------------------------------------------------
3. // Constants
4. // ----------------------------------------------------------------------------------
5. // Version                   : 0.9.1
6. // Authors                   : Chris Bizer (chris@bizer.de),
7. //                             Daniel Westphal (dawe@gmx.de),
8. //                               Leandro Mariano Lopez (llopez@xinergiaargentina.com),
9. //                             Radoslaw Oldakowski (radol@gmx.de)
10. //
11. // Description               : Constants and default configuration
12. // ----------------------------------------------------------------------------------
13. // History:
14. // 08-10-2004                 : UNIC_RDF added
15. // 08-09-2004                  : PACKAGES added
16. // 06-13-2004                 : INDEX_TYPE added
17. // 11-27-2003                  : N3SER_BNODE_SHORT added
18. // 11-18-2003                  : RDF_PARSE_TYPE_COLLECTION, RDF_XMLLITERAL,
19. //                                IN_PROPERTY_PARSE_TYPE_COLLECTION, VALIDATE_IDS added.
20. // 11-13-2003                  : HIDE_ADVERTISE added
21. // 11-12-2003                  : FIX_BLANKNODES added
22. // 07-27-2003                 : Database, RDQL Error Messages,
23. //                              RDQL default namespace prefixes added
24. // 02-12-2003                  : XML_NAMESPACE_DECLARATION_PREFIX changed.
25. // 01-15-2003                  : Some syntax corrections to avoid PHP notices added.
26. // 01-10-2003                 : Constants RDF_NODEID, RDF_SEEALSO, RDF_OBJECT_TYPE_BNODE,
27. //                              RDF_SUBJECT_TYPE_BNODE added
28. // 12-18-2002                 : RDF_DATATYPE, $short_datatype added
29. // 10-03-2002                 : Some RDF Shema constants added
30. // 09-15-2002                 : Initial version
31. // ----------------------------------------------------------------------------------


34. // ----------------------------------------------------------------------------------
35. // General
36. // ----------------------------------------------------------------------------------


40. define('RDFAPI_ERROR', 'RDFAPI error ');
41. define('DEFAULT_ALGORITHM', 'MD5');
42. define('DEFAULT_ENCODING', 'UTF-8');
43. define('INDENTATION', '   ');
44. define('LINEFEED', chr(10));

46. // ----------------------------------------------------------------------------------
47. // RAP Packages
48. // ----------------------------------------------------------------------------------

50. define('PACKAGE_MODEL','model/ModelP.php');
51. define('PACKAGE_UTILITY','util/Utility.php');
52. define('PACKAGE_DBASE','model/DBase.php');
53. define('PACKAGE_SYNTAX_RDF','syntax/SyntaxRDF.php');
54. define('PACKAGE_SYNTAX_N3','syntax/SyntaxN3.php');
55. define('PACKAGE_SYNTAX_GRDDL','syntax/SyntaxGRDDL.php');
56. define('PACKAGE_VOCABULARY','vocabulary/Vocabulary.php');
57. define('PACKAGE_RDQL','rdql/RDQL.php');
58. define('PACKAGE_INFMODEL','infModel/InfModelP.php');
59. define('PACKAGE_RESMODEL','resModel/ResModelP.php');
60. define('PACKAGE_ONTMODEL','ontModel/OntModelP.php');

62. // ----------------------------------------------------------------------------------
63. // Model
64. // ----------------------------------------------------------------------------------

66. // Defines a prefix used in the ID of automatically created bNodes.

68. define('BNODE_PREFIX', 'bNode');

70. // Sets the index of MemModell:
71. // IND_DEF: Defaultindex over subject, predicate, obeject seperate.
72. // IND_SPO: Index over subject+predicate+object.
73. // IND_SP:  Index over subject+predicate.
74. // IND_SO:  Index over subject+object.

76. define('NO_INDEX',-1);
77. define('IND_DEF',0);
78. define('IND_SPO',1);
79. define('IND_SP',2);
80. define('IND_SO',3);
81. define('INDEX_TYPE',IND_DEF);

83. // ----------------------------------------------------------------------------------
84. // ModelFactory
85. // ----------------------------------------------------------------------------------


89. define ('MEMMODEL','MemModel');
90. define ('DBMODEL','DbModel');
91. define ('INFMODELF','InfModelF');
92. define ('INFMODELB','InfModelB');
93. define ('ONTMODEL','OntModel');
94. define ('RESMODEL','ResModel');
95. define ('RDFS_VOCABULARY','RdfsVocabulary.php');

97. // ----------------------------------------------------------------------------------
98. // Parser
99. // ----------------------------------------------------------------------------------

101. // RdfParser: Set this option to false if you want to use IDs containing CombiningChars or
102. // Extenders (see http://www.w3.org/TR/REC-xml-names/#NT-NCName). If set to TRUE, they're assumed to be invalid.

104. define('VALIDATE_IDS', TRUE);

106. // RdfParser: Set this option to true if you want to parse UNICODE documents.
107. // WARNING: Setting the option TRUE significantly slows down the RDF-parser.

109. define('UNIC_RDF', TRUE);

111. // RdfParser: Set this option to true if you want to make sure that the created RDF-model doesnt contain
112. // duplicate RDF-statements. WARNING: Setting the option TRUE significantly slows down the RDF-parser.

114. define('CREATE_MODEL_WITHOUT_DUPLICATES', FALSE);

116. // N3 and N-Triple-Parser: Set this option to true in order to override the given bnode
117. // labels and rename them to the defined BNODE_PREFIX

119. define('FIX_BLANKNODES', TRUE);

121. define('NAMESPACE_SEPARATOR_CHAR','^');
122. define('NAMESPACE_SEPARATOR_STRING','^');
123. define('IN_TOP_LEVEL',0);
124. define('IN_RDF',1);
125. define('IN_DESCRIPTION',2);
126. define('IN_PROPERTY_UNKNOWN_OBJECT',3);
127. define('IN_PROPERTY_RESOURCE',4);
128. define('IN_PROPERTY_EMPTY_RESOURCE',5);
129. define('IN_PROPERTY_LITERAL',6);
130. define('IN_PROPERTY_PARSE_TYPE_LITERAL',7);
131. define('IN_PROPERTY_PARSE_TYPE_RESOURCE',8);
132. define('IN_XML',9);
133. define('IN_UNKNOWN',10);
134. define('IN_PROPERTY_PARSE_TYPE_COLLECTION', 11);
135. define('RDF_SUBJECT_TYPE_URI',0);
136. define('RDF_SUBJECT_TYPE_DISTRIBUTED',1);
137. define('RDF_SUBJECT_TYPE_PREFIX',2);
138. define('RDF_SUBJECT_TYPE_ANONYMOUS',3);
139. define('RDF_SUBJECT_TYPE_BNODE',4);
140. define('RDF_OBJECT_TYPE_RESOURCE',0);
141. define('RDF_OBJECT_TYPE_LITERAL',1);
142. define('RDF_OBJECT_TYPE_XML',2);
143. define('RDF_OBJECT_TYPE_BNODE',3);

145. // ----------------------------------------------------------------------------------
146. // Serializer
147. // ----------------------------------------------------------------------------------

149. // RDF, N3, N-Triple Serializer: set to TRUE in oder to suppres the "Generated by RAP"
150. // comment in the output files.

152. define('HIDE_ADVERTISE',FALSE);

154. // RDF Serializer: Set to TRUE, if the serializer should use entities for URIs.
155. define('SER_USE_ENTITIES', FALSE );

157. // RDF Serializer: Set to TRUE, if the serializer should serialize triples as XML
158. // attributes where possible.

160. define('SER_USE_ATTRIBUTES', FALSE );

162. // RDF Serializer: Set to TRUE in order to sort the statements of a model before
163. // serializing them.

165. define('SER_SORT_MODEL', FALSE );


169. // RDF Serializer: Set to TRUE, if the serializer should use qualified names for RDF
170. // reserved words.
171. // NOTE: There is only one default namespace allowed within an XML document.
172. //       Therefore if SER_RDF_QNAMES in is set to FALSE and you pass the parameter
173. //       $xml_default_namespace to the method serialize() of class RdfSerializer,
174. //       the model will be serialized as if SER_RDF_QNAMES were set to TRUE.

176. define('SER_RDF_QNAMES', TRUE );

178. // RDF Serializer: Set to TRUE, if the serializer should start documents with the
179. // xml declaration <?xml version="1.0" encoding="UTF-8" >.

181. define('SER_XML_DECLARATION', TRUE );

183. // N3 Serializer: Set to TRUE, if the N3 serializer should try to compress the blank node
184. // syntax using [] whereever possible.

186. define('N3SER_BNODE_SHORT', FALSE);

188. // RDF Serializer: Set to TRUE, if the serializer should write text values always as
189. // escaped CDATA.

191. define('USE_CDATA', FALSE);

193. define('USE_ANY_QUOTE', FALSE);
194. define('GENERAL_PREFIX_BASE','ns');
195. define('MAX_ALLOWED_ABBREVIATED_LENGTH',60);

197. // ----------------------------------------------------------------------------------
198. // Util
199. // ----------------------------------------------------------------------------------

201. // Definition of the colors used by the method RDFUtil:writeHTMLTable

203. define('HTML_TABLE_HEADER_COLOR', '#FFFFFF');
204. define('HTML_TABLE_RESOURCE_COLOR', '#FFFFCC');
205. define('HTML_TABLE_LITERAL_COLOR', '#E7E7EF');
206. define('HTML_TABLE_BNODE_COLOR', '#FFCCFF');
207. define('HTML_TABLE_RDF_NS_COLOR', '#CCFFCC');
208. define('HTML_TABLE_NS_ROW_COLOR1', '#FFFFFF');
209. define('HTML_TABLE_NS_ROW_COLOR0', '#E7E7EF');

211. // ----------------------------------------------------------------------------------
212. // RDF
213. // ----------------------------------------------------------------------------------


217. define('RDF_NAMESPACE_URI','http://www.w3.org/1999/02/22-rdf-syntax-ns#' );
218. define('RDF_NAMESPACE_PREFIX','rdf' );
219. define('RDF_RDF','RDF');
220. define('RDF_DESCRIPTION','Description');
221. define('RDF_ID','ID');
222. define('RDF_ABOUT','about');
223. define('RDF_ABOUT_EACH','aboutEach');
224. define('RDF_ABOUT_EACH_PREFIX','aboutEachPrefix');
225. define('RDF_BAG_ID','bagID');
226. define('RDF_RESOURCE','resource');
227. define('RDF_VALUE','value');
228. define('RDF_PARSE_TYPE','parseType');
229. define('RDF_PARSE_TYPE_LITERAL','Literal');
230. define('RDF_PARSE_TYPE_RESOURCE','Resource');
231. define('RDF_PARSE_TYPE_COLLECTION', 'Collection');
232. define('RDF_TYPE','type');
233. define('RDF_BAG','Bag');
234. define('RDF_SEQ','Seq');
235. define('RDF_ALT','Alt');
236. define('RDF_LI','li');
237. define('RDF_STATEMENT','Statement');
238. define('RDF_SUBJECT','subject');
239. define('RDF_PREDICATE','predicate');
240. define('RDF_OBJECT','object');
241. define('RDF_NODEID','nodeID');
242. define('RDF_DATATYPE','datatype');
243. define('RDF_SEEALSO','seeAlso');
244. define('RDF_PROPERTY','Property');
245. define('RDF_LIST','List');
246. define('RDF_NIL','nil');
247. define('RDF_REST','rest');
248. define('RDF_FIRST','first');
249. define('RDF_XMLLITERAL', 'XMLLiteral');

251. // ----------------------------------------------------------------------------------
252. // RDF Schema
253. // ----------------------------------------------------------------------------------


257. define('RDF_SCHEMA_URI','http://www.w3.org/2000/01/rdf-schema#' );
258. define('RDF_DATATYPE_SCHEMA_URI','http://www.w3.org/TR/xmlschema-2' );
259. define('RDF_SCHEMA_PREFIX', 'rdfs');
260. define('RDFS_SUBCLASSOF','subClassOf');
261. define('RDFS_SUBPROPERTYOF','subPropertyOf');
262. define('RDFS_RANGE','range');
263. define('RDFS_DOMAIN','domain');
264. define('RDFS_CLASS','Class');
265. define('RDFS_RESOURCE','Resource');
266. define('RDFS_DATATYPE','Datatype');
267. define('RDFS_LITERAL','Literal');
268. define('RDFS_SEE_ALSO','seeAlso');
269. define('RDFS_IS_DEFINED_BY','isDefinedBy');
270. define('RDFS_LABEL','label');
271. define('RDFS_COMMENT','comment');


274. // ----------------------------------------------------------------------------------
275. // OWL
276. // ----------------------------------------------------------------------------------


280. define('OWL_URI','http://www.w3.org/2002/07/owl#' );
281. define('OWL_PREFIX', 'owl');
282. define('OWL_SAME_AS','sameAs');
283. define('OWL_INVERSE_OF','inverseOf');


286. // ----------------------------------------------------------------------------------
287. // XML
288. // ----------------------------------------------------------------------------------


292. define('XML_NAMESPACE_PREFIX', 'xml');
293. define('XML_NAMESPACE_DECLARATION_PREFIX', 'xmlns');
294. define('XML_NAMESPACE_URI','http://www.w3.org/XML/1998/namespace' );
295. define('XML_LANG','lang');
296. define('DATATYPE_SHORTCUT_PREFIX','datatype:');

298. // ----------------------------------------------------------------------------------
299. // RDF DATATYPE SHORTCUTS (extends datatype shortcuts to the full XML datatype URIs)
300. // ----------------------------------------------------------------------------------


304. $short_datatype = array(
305. 'STRING'    => RDF_DATATYPE_SCHEMA_URI . '#string',
306. 'DECIMAL'   => RDF_DATATYPE_SCHEMA_URI . '#decimal',
307. 'INTEGER'   => RDF_DATATYPE_SCHEMA_URI . '#integer',
308. 'INT'       => RDF_DATATYPE_SCHEMA_URI . '#int',
309. 'SHORT'     => RDF_DATATYPE_SCHEMA_URI . '#short',
310. 'BYTE'      => RDF_DATATYPE_SCHEMA_URI . '#byte',
311. 'LONG'      => RDF_DATATYPE_SCHEMA_URI . '#long',
312. 'LANGUAGE'  => RDF_DATATYPE_SCHEMA_URI . '#language',
313. 'NAME'      => RDF_DATATYPE_SCHEMA_URI . '#name'
314. );

316. // ----------------------------------------------------------------------------------
317. // Database
318. // ----------------------------------------------------------------------------------


322. define('ADODB_DB_DRIVER', 'ODBC');
323. define('ADODB_DB_HOST', 'rap');
324. define('ADODB_DB_NAME', '');
325. define('ADODB_DB_USER', '');
326. define('ADODB_DB_PASSWORD', '');
327. define('ADODB_DEBUG_MODE', '0');


330. // ----------------------------------------------------------------------------------
331. // RDQL Error Messages
332. // ----------------------------------------------------------------------------------


336. define('RDQL_ERR','RDQL error ');
337. define('RDQL_SYN_ERR','RDQL syntax error ');
338. define('RDQL_SEL_ERR', RDQL_ERR .'in the SELECT clause: ');
339. define('RDQL_SRC_ERR', RDQL_ERR .'in the SOURCE clause: ');
340. define('RDQL_WHR_ERR', RDQL_ERR .'in the WHERE clause: ');
341. define('RDQL_AND_ERR', RDQL_ERR .'in the AND clause: ');
342. define('RDQL_USG_ERR', RDQL_ERR .'in the USING clause: ');


345. // ----------------------------------------------------------------------------------
346. // Vocabulary
347. // ----------------------------------------------------------------------------------
348. // namespace declarations

350. define('ATOM_NS', 'http://purl.org/atom/ns#');
351. define('DC_NS', 'http://purl.org/dc/elements/1.1/');
352. define('DCTERM_NS', 'http://purl.org/dc/terms/');
353. define('DCMITYPE_NS', 'http://purl.org/dc/dcmitype/');
354. define('FOAF_NS', 'http://xmlns.com/foaf/0.1/#');
355. define('OWL_NS', 'http://www.w3.org/2002/07/owl#');
356. define('RSS_NS', 'http://purl.org/rss/1.0/#');
357. define('VCARD_NS', 'http://www.w3.org/2001/vcard-rdf/3.0#');


361. // ----------------------------------------------------------------------------------
362. // RDQL and parser default namespace prefixes
363. // ----------------------------------------------------------------------------------


367. $default_prefixes = array(
368. XML_NAMESPACE_PREFIX => XML_NAMESPACE_URI,
369. RDF_NAMESPACE_PREFIX => RDF_NAMESPACE_URI,
370. RDF_SCHEMA_PREFIX => RDF_SCHEMA_URI,
371. 'xsd'  => 'http://www.w3.org/2001/XMLSchema#',
372. OWL_PREFIX => OWL_URI
373. );

375. // ----------------------------------------------------------------------------------
376. // InfModel
377. // ----------------------------------------------------------------------------------

379. //activate / deactivate reasoning for the following schema constructs
380. //rdfs:subclass

382. define('INF_RES_SUBCLASSOF',true);
383. //rdfs:subproperty
384. define('INF_RES_SUBPROPERTYOF',true);
385. //rdfs:range
386. define('INF_RES_RANGE',true);
387. //rdfs:domain
388. define('INF_RES_DOMAIN',true);
389. //owl:sameAs
390. define('INF_RES_OWL_SAMEAS',true);
391. //owl:inverseOf
392. define('INF_RES_OWL_INVERSEOF',true);

394. //generic RDFS Rules from the RDF Schema doc:
395. //see: http://www.w3.org/TR/2004/REC-rdf-mt-20040210/#RDFSRules

397. define('INF_RES_RULE_RDFS12',false);
398. define('INF_RES_RULE_RDFS6',false);
399. define('INF_RES_RULE_RDFS8',false);
400. define('INF_RES_RULE_RDFS10',false);
401. define('INF_RES_RULE_RDFS13',false);


406. ?>
```

Documentation generated on Fri, 17 Dec 2004 16:14:27 +0100 by phpDocumentor 1.3.0RC3
